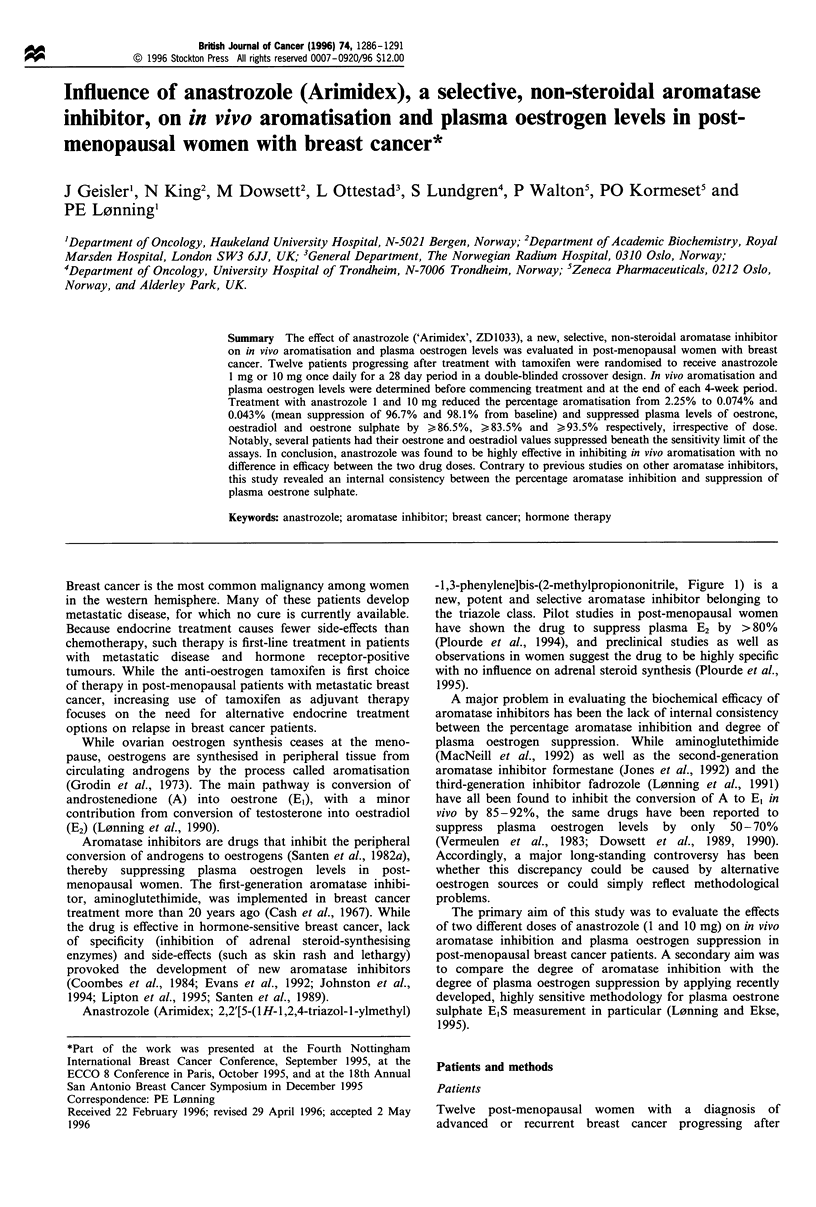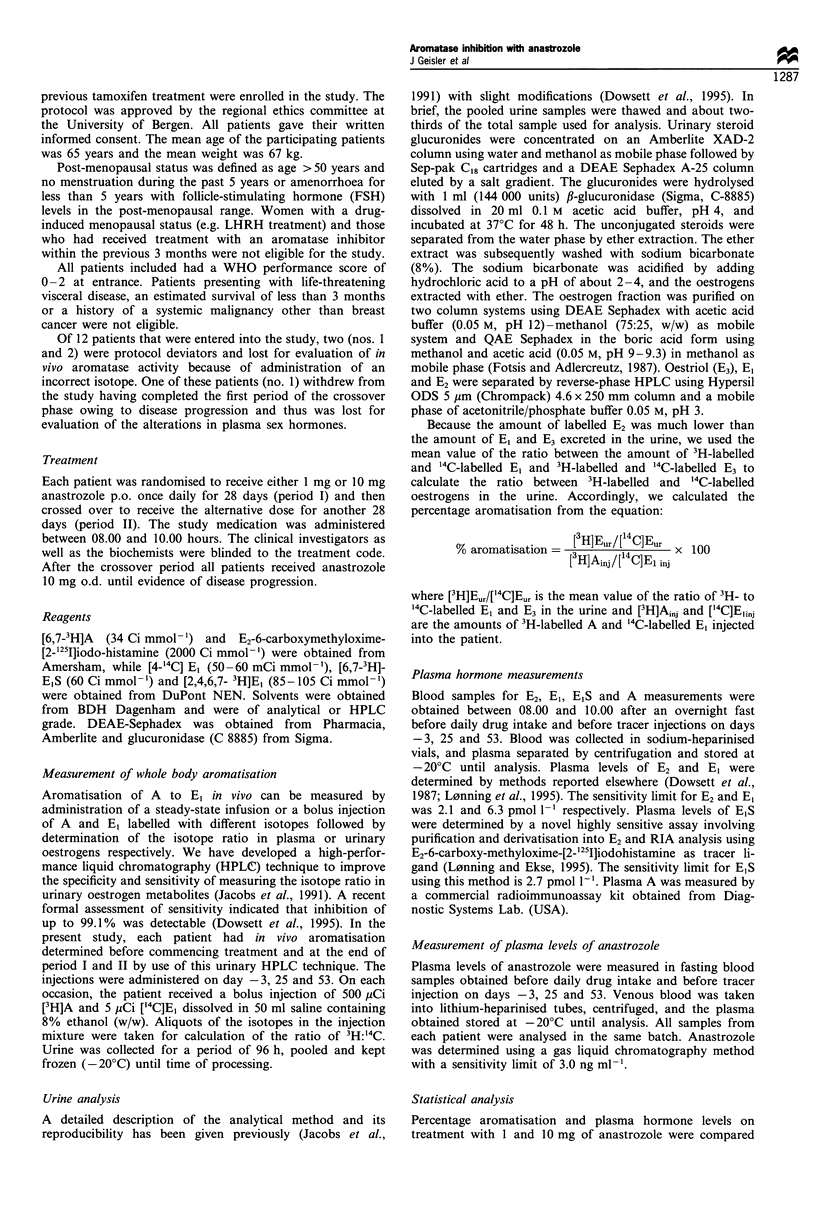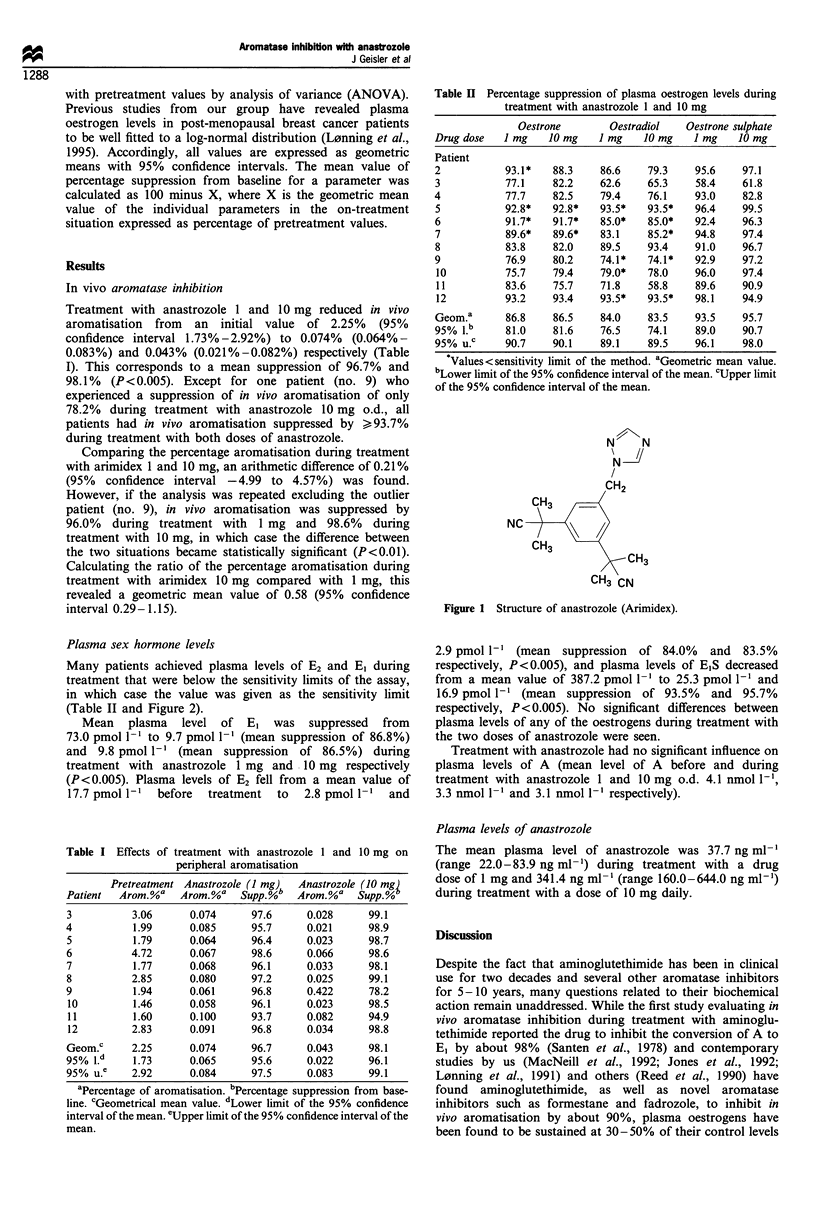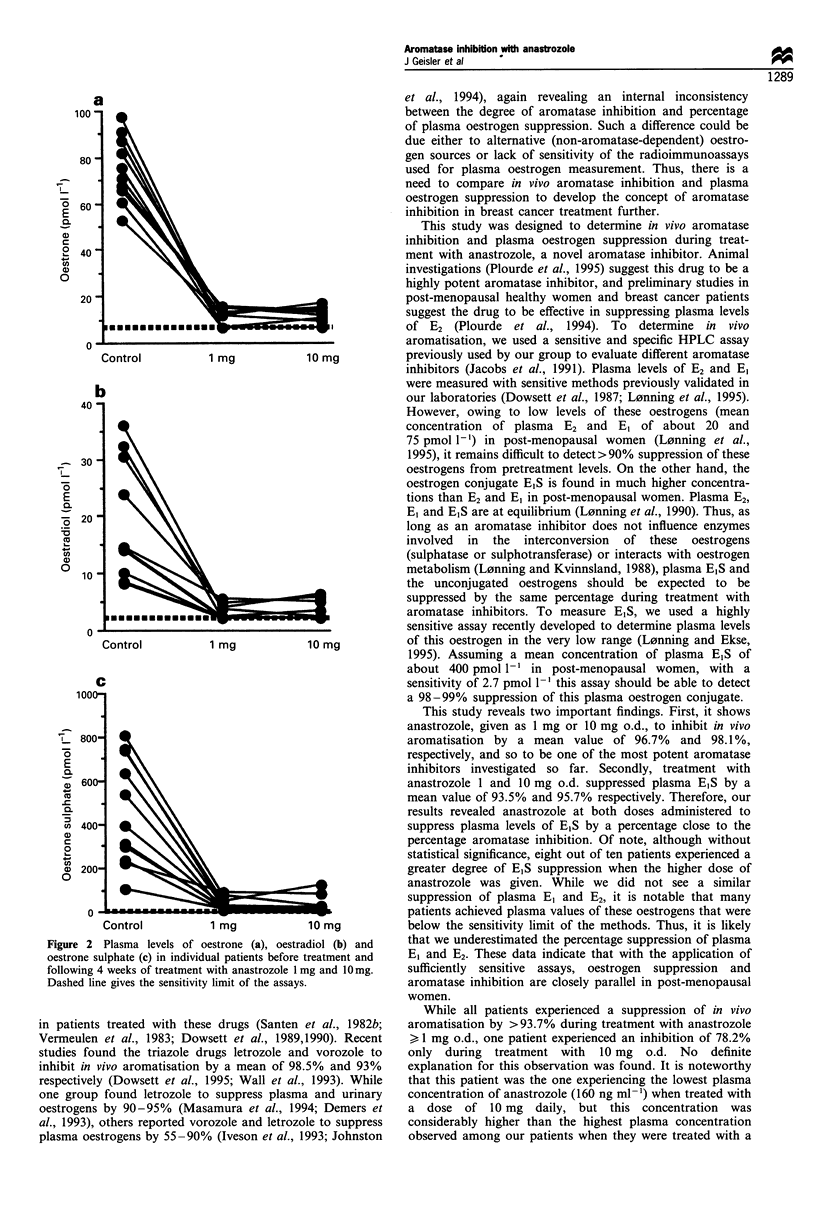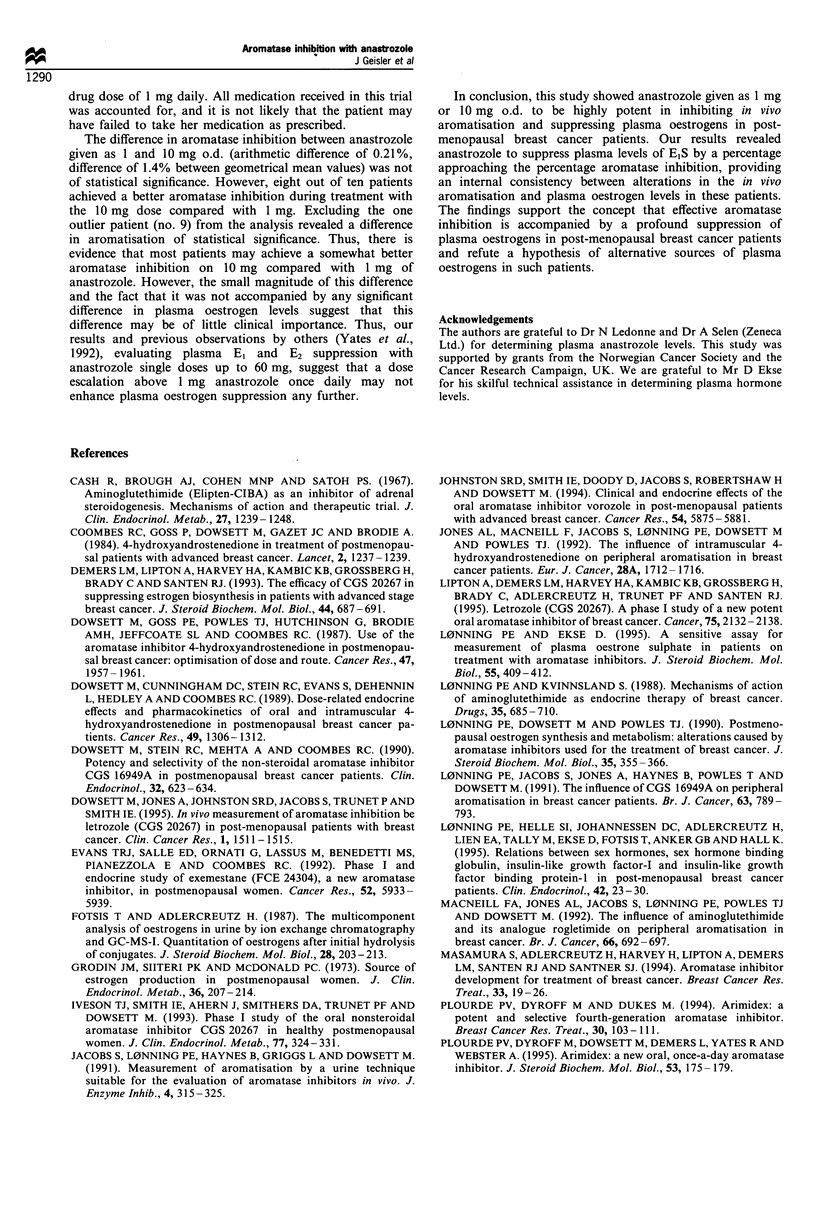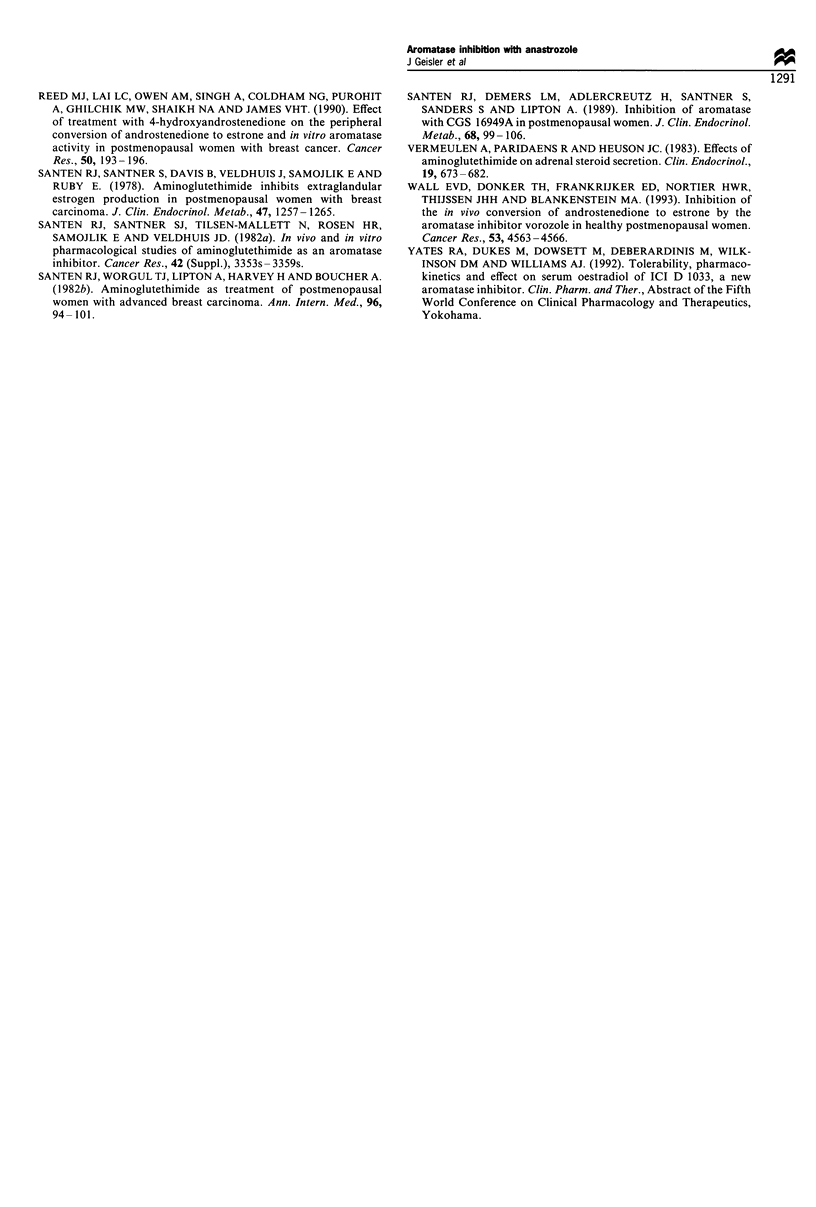# Influence of anastrozole (Arimidex), a selective, non-steroidal aromatase inhibitor, on in vivo aromatisation and plasma oestrogen levels in postmenopausal women with breast cancer.

**DOI:** 10.1038/bjc.1996.531

**Published:** 1996-10

**Authors:** J. Geisler, N. King, M. Dowsett, L. Ottestad, S. Lundgren, P. Walton, P. O. Kormeset, P. E. Lønning

**Affiliations:** Department of Oncology, Haukeland University Hospital, Bergen, Norway.

## Abstract

The effect of anastrozole ('Arimidex', ZD1033), a new, selective, non-steroidal aromatase inhibitor on in vivo aromatisation and plasma oestrogen levels was evaluated in post-menopausal women with breast cancer. Twelve patients progressing after treatment with tamoxifen were randomised to receive anastrozole 1 mg or 10 mg once daily for a 28 day period in a double-blinded crossover design. In vivo aromatisation and plasma oestrogen levels were determined before commencing treatment and at the end of each 4-week period. Treatment with anastrozole 1 and 10 mg reduced the percentage aromatisation from 2.25% to 0.074% and 0.043% (mean suppression of 96.7% and 98.1% from baseline) and suppressed plasma levels of oestrone, oestradiol and oestrone sulphate by > or = 86.5%, > or = 83.5% and > or = 93.5% respectively, irrespective of dose. Notably, several patients had their oestrone and oestradiol values suppressed beneath the sensitivity limit of the assays. In conclusion, anastrozole was found to be highly effective in inhibiting in vivo aromatisation with no difference in efficacy between the two drug doses. Contrary to previous studies on other aromatase inhibitors, this study revealed an internal consistency between the percentage aromatase inhibition and suppression of plasma oestrone sulphate.


British Journal of Cancer (1996) 74, 1286-1291
© 1996 Stockton Press All rights reserved 0007-0920/96 $12.00

### Influence of anastrozole (Arimidex), a selective, non-steroidal aromatase inhibitor, on *in vivo* aromatisation and plasma oestrogen levels in post-menopausal women with breast cancer*

J Geisler[1], N King[2], M Dowsett[2], L Ottestad[3], S Lundgren[4], P Walton[5], PO Kormeset[5] and PE Lønning[1]

[1]*Department of Oncology, Haukeland University Hospital, N-5021 Bergen, Norway;* [2]*Department of Academic Biochemistry, Royal Marsden Hospital, London SW3 6JJ, UK;* [3]*General Department, The Norwegian Radium Hospital, 0310 Oslo, Norway;* [4]*Department of Oncology, University Hospital of Trondheim, N-7006 Trondheim, Norway;* [5]*Zeneca Pharmaceuticals, 0212 Oslo, Norway, and Alderley Park, UK.*

**Summary** The effect of anastrozole ('Arimidex', ZD1033), a new, selective, non-steroidal aromatase inhibitor on *in vivo* aromatisation and plasma oestrogen levels was evaluated in post-menopausal women with breast cancer. Twelve patients progressing after treatment with tamoxifen were randomised to receive anastrozole 1 mg or 10 mg once daily for a 28 day period in a double-blinded crossover design. *In vivo* aromatisation and plasma oestrogen levels were determined before commencing treatment and at the end of each 4-week period. Treatment with anastrozole 1 and 10 mg reduced the percentage aromatisation from 2.25% to 0.074% and 0.043% (mean suppression of 96.7% and 98.1% from baseline) and suppressed plasma levels of oestrone, oestradiol and oestrone sulphate by ⩾86.5%, ⩾83.5% and ⩾93.5% respectively, irrespective of dose. Notably, several patients had their oestrone and oestradiol values suppressed beneath the sensitivity limit of the assays. In conclusion, anastrozole was found to be highly effective in inhibiting *in vivo* aromatisation with no difference in efficacy between the two drug doses. Contrary to previous studies on other aromatase inhibitors, this study revealed an internal consistency between the percentage aromatase inhibition and suppression of plasma oestrone sulphate.

**Keywords:** anastrozole; aromatase inhibitor; breast cancer; hormone therapy

Breast cancer is the most common malignancy among women in the western hemisphere. Many of these patients develop metastatic disease, for which no cure is currently available. Because endocrine treatment causes fewer side-effects than chemotherapy, such therapy is first-line treatment in patients with metastatic disease and hormone receptor-positive tumours. While the anti-oestrogen tamoxifen is first choice of therapy in post-menopausal patients with metastatic breast cancer, increasing use of tamoxifen as adjuvant therapy focuses on the need for alternative endocrine treatment options on relapse in breast cancer patients.

While ovarian oestrogen synthesis ceases at the menopause, oestrogens are synthesised in peripheral tissue from circulating androgens by the process called aromatisation (Grodin *et al.*, 1973). The main pathway is conversion of androstenedione (A) into oestrone ($E_1$), with a minor contribution from conversion of testosterone into oestradiol ($E_2$) (Lønning *et al.*, 1990).

Aromatase inhibitors are drugs that inhibit the peripheral conversion of androgens to oestrogens (Santen *et al.*, 1982*a*), thereby suppressing plasma oestrogen levels in post-menopausal women. The first-generation aromatase inhibitor, aminoglutethimide, was implemented in breast cancer treatment more than 20 years ago (Cash *et al.*, 1967). While the drug is effective in hormone-sensitive breast cancer, lack of specificity (inhibition of adrenal steroid-synthesising enzymes) and side-effects (such as skin rash and lethargy) provoked the development of new aromatase inhibitors (Coombes *et al.*, 1984; Evans *et al.*, 1992; Johnston *et al.*, 1994; Lipton *et al.*, 1995; Santen *et al.*, 1989).

Anastrozole (Arimidex; 2,2'[5-(1*H*-1,2,4-triazol-1-ylmethyl)-1,3-phenylene]bis-(2-methylpropiononitrile, Figure 1) is a new, potent and selective aromatase inhibitor belonging to the triazole class. Pilot studies in post-menopausal women have shown the drug to suppress plasma $E_2$ by >80% (Plourde *et al.*, 1994), and preclinical studies as well as observations in women suggest the drug to be highly specific with no influence on adrenal steroid synthesis (Plourde *et al.*, 1995).

A major problem in evaluating the biochemical efficacy of aromatase inhibitors has been the lack of internal consistency between the percentage aromatase inhibition and degree of plasma oestrogen suppression. While aminoglutethimide (MacNeill *et al.*, 1992) as well as the second-generation aromatase inhibitor formestane (Jones *et al.*, 1992) and the third-generation inhibitor fadrozole (Lønning *et al.*, 1991) have all been found to inhibit the conversion of A to $E_1$ *in vivo* by 85–92%, the same drugs have been reported to suppress plasma oestrogen levels by only 50–70% (Vermeulen *et al.*, 1983; Dowsett *et al.*, 1989, 1990). Accordingly, a major long-standing controversy has been whether this discrepancy could be caused by alternative oestrogen sources or could simply reflect methodological problems.

The primary aim of this study was to evaluate the effects of two different doses of anastrozole (1 and 10 mg) on *in vivo* aromatase inhibition and plasma oestrogen suppression in post-menopausal breast cancer patients. A secondary aim was to compare the degree of aromatase inhibition with the degree of plasma oestrogen suppression by applying recently developed, highly sensitive methodology for plasma oestrone sulphate $E_1S$ measurement in particular (Lønning and Ekse, 1995).

### Patients and methods

### *Patients*

Twelve post-menopausal women with a diagnosis of advanced or recurrent breast cancer progressing after

*Part of the work was presented at the Fourth Nottingham International Breast Cancer Conference, September 1995, at the ECCO 8 Conference in Paris, October 1995, and at the 18th Annual San Antonio Breast Cancer Symposium in December 1995

Correspondence: PE Lønning

Received 22 February 1996; revised 29 April 1996; accepted 2 May 1996

previous tamoxifen treatment were enrolled in the study. The protocol was approved by the regional ethics committee at the University of Bergen. All patients gave their written informed consent. The mean age of the participating patients was 65 years and the mean weight was 67 kg.

Post-menopausal status was defined as age >50 years and no menstruation during the past 5 years or amenorrhoea for less than 5 years with follicle-stimulating hormone (FSH) levels in the post-menopausal range. Women with a drug-induced menopausal status (e.g. LHRH treatment) and those who had received treatment with an aromatase inhibitor within the previous 3 months were not eligible for the study.

All patients included had a WHO performance score of 0–2 at entrance. Patients presenting with life-threatening visceral disease, an estimated survival of less than 3 months or a history of a systemic malignancy other than breast cancer were not eligible.

Of 12 patients that were entered into the study, two (nos. 1 and 2) were protocol deviators and lost for evaluation of *in vivo* aromatase activity because of administration of an incorrect isotope. One of these patients (no. 1) withdrew from the study having completed the first period of the crossover phase owing to disease progression and thus was lost for evaluation of the alterations in plasma sex hormones.

### *Treatment*

Each patient was randomised to receive either 1 mg or 10 mg anastrozole p.o. once daily for 28 days (period I) and then crossed over to receive the alternative dose for another 28 days (period II). The study medication was administered between 08.00 and 10.00 hours. The clinical investigators as well as the biochemists were blinded to the treatment code. After the crossover period all patients received anastrozole 10 mg o.d. until evidence of disease progression.

### *Reagents*

[6,7-$^3$H]A (34 Ci mmol$^{-1}$) and $E_2$-6-carboxymethyloxime-[2-$^{125}$I]iodo-histamine (2000 Ci mmol$^{-1}$) were obtained from Amersham, while [4-$^{14}$C] $E_1$ (50–60 mCi mmol$^{-1}$), [6,7-$^3$H]-$E_1$S (60 Ci mmol$^{-1}$) and [2,4,6,7- $^3$H]$E_1$ (85–105 Ci mmol$^{-1}$) were obtained from DuPont NEN. Solvents were obtained from BDH Dagenham and were of analytical or HPLC grade. DEAE-Sephadex was obtained from Pharmacia, Amberlite and glucuronidase (C 8885) from Sigma.

### *Measurement of whole body aromatisation*

Aromatisation of A to $E_1$ *in vivo* can be measured by administration of a steady-state infusion or a bolus injection of A and $E_1$ labelled with different isotopes followed by determination of the isotope ratio in plasma or urinary oestrogens respectively. We have developed a high-performance liquid chromatography (HPLC) technique to improve the specificity and sensitivity of measuring the isotope ratio in urinary oestrogen metabolites (Jacobs *et al.*, 1991). A recent formal assessment of sensitivity indicated that inhibition of up to 99.1% was detectable (Dowsett *et al.*, 1995). In the present study, each patient had *in vivo* aromatisation determined before commencing treatment and at the end of period I and II by use of this urinary HPLC technique. The injections were administered on day −3, 25 and 53. On each occasion, the patient received a bolus injection of 500 $\mu$Ci [$^3$H]A and 5 $\mu$Ci [$^{14}$C]$E_1$ dissolved in 50 ml saline containing 8% ethanol (w/w). Aliquots of the isotopes in the injection mixture were taken for calculation of the ratio of $^3$H:$^{14}$C. Urine was collected for a period of 96 h, pooled and kept frozen (−20°C) until time of processing.

### *Urine analysis*

A detailed description of the analytical method and its reproducibility has been given previously (Jacobs *et al.*, 1991) with slight modifications (Dowsett *et al.*, 1995). In brief, the pooled urine samples were thawed and about two-thirds of the total sample used for analysis. Urinary steroid glucuronides were concentrated on an Amberlite XAD-2 column using water and methanol as mobile phase followed by Sep-pak $C_{18}$ cartridges and a DEAE Sephadex A-25 column eluted by a salt gradient. The glucuronides were hydrolysed with 1 ml (144 000 units) $\beta$-glucuronidase (Sigma, C-8885) dissolved in 20 ml 0.1 M acetic acid buffer, pH 4, and incubated at 37°C for 48 h. The unconjugated steroids were separated from the water phase by ether extraction. The ether extract was subsequently washed with sodium bicarbonate (8%). The sodium bicarbonate was acidified by adding hydrochloric acid to a pH of about 2–4, and the oestrogens extracted with ether. The oestrogen fraction was purified on two column systems using DEAE Sephadex with acetic acid buffer (0.05 M, pH 12)–methanol (75:25, w/w) as mobile system and QAE Sephadex in the boric acid form using methanol and acetic acid (0.05 M, pH 9–9.3) in methanol as mobile phase (Fotsis and Adlercreutz, 1987). Oestriol ($E_3$), $E_1$ and $E_2$ were separated by reverse-phase HPLC using Hypersil ODS 5 $\mu$m (Chrompack) 4.6 × 250 mm column and a mobile phase of acetonitrile/phosphate buffer 0.05 M, pH 3.

Because the amount of labelled $E_2$ was much lower than the amount of $E_1$ and $E_3$ excreted in the urine, we used the mean value of the ratio between the amount of $^3$H-labelled and $^{14}$C-labelled $E_1$ and $^3$H-labelled and $^{14}$C-labelled $E_3$ to calculate the ratio between $^3$H-labelled and $^{14}$C-labelled oestrogens in the urine. Accordingly, we calculated the percentage aromatisation from the equation:

$$\%\ \text{aromatisation} = \frac{[^3\text{H}]\text{E}_{\text{ur}}/[^{14}\text{C}]\text{E}_{\text{ur}}}{[^3\text{H}]\text{A}_{\text{inj}}/[^{14}\text{C}]\text{E}_{1\ \text{inj}}} \times 100$$

where [$^3$H]$E_{ur}$/[$^{14}$C]$E_{ur}$ is the mean value of the ratio of $^3$H- to $^{14}$C-labelled $E_1$ and $E_3$ in the urine and [$^3$H]$A_{inj}$ and [$^{14}$C]$E_{1inj}$ are the amounts of $^3$H-labelled A and $^{14}$C-labelled $E_1$ injected into the patient.

### *Plasma hormone measurements*

Blood samples for $E_2$, $E_1$, $E_1$S and A measurements were obtained between 08.00 and 10.00 after an overnight fast before daily drug intake and before tracer injections on days −3, 25 and 53. Blood was collected in sodium-heparinised vials, and plasma separated by centrifugation and stored at −20°C until analysis. Plasma levels of $E_2$ and $E_1$ were determined by methods reported elsewhere (Dowsett *et al.*, 1987; Lønning *et al.*, 1995). The sensitivity limit for $E_2$ and $E_1$ was 2.1 and 6.3 pmol l$^{-1}$ respectively. Plasma levels of $E_1$S were determined by a novel highly sensitive assay involving purification and derivatisation into $E_2$ and RIA analysis using $E_2$-6-carboxy-methyloxime-[2-$^{125}$I]iodohistamine as tracer ligand (Lønning and Ekse, 1995). The sensitivity limit for $E_1$S using this method is 2.7 pmol l$^{-1}$. Plasma A was measured by a commercial radioimmunoassay kit obtained from Diagnostic Systems Lab. (USA).

### *Measurement of plasma levels of anastrozole*

Plasma levels of anastrozole were measured in fasting blood samples obtained before daily drug intake and before tracer injection on days −3, 25 and 53. Venous blood was taken into lithium-heparinised tubes, centrifuged, and the plasma obtained stored at −20°C until analysis. All samples from each patient were analysed in the same batch. Anastrozole was determined using a gas liquid chromatography method with a sensitivity limit of 3.0 ng ml$^{-1}$.

### *Statistical analysis*

Percentage aromatisation and plasma hormone levels on treatment with 1 and 10 mg of anastrozole were compared with pretreatment values by analysis of variance (ANOVA). Previous studies from our group have revealed plasma oestrogen levels in post-menopausal breast cancer patients to be well fitted to a log-normal distribution (Lønning *et al.*, 1995). Accordingly, all values are expressed as geometric means with 95% confidence intervals. The mean value of percentage suppression from baseline for a parameter was calculated as 100 minus X, where X is the geometric mean value of the individual parameters in the on-treatment situation expressed as percentage of pretreatment values.

### Results

### In vivo *aromatase inhibition*

Treatment with anastrozole 1 and 10 mg reduced *in vivo* aromatisation from an initial value of 2.25% (95% confidence interval 1.73%–2.92%) to 0.074% (0.064%–0.083%) and 0.043% (0.021%–0.082%) respectively (Table I). This corresponds to a mean suppression of 96.7% and 98.1% ($P<0.005$). Except for one patient (no. 9) who experienced a suppression of *in vivo* aromatisation of only 78.2% during treatment with anastrozole 10 mg o.d., all patients had *in vivo* aromatisation suppressed by ⩾93.7% during treatment with both doses of anastrozole.

Comparing the percentage aromatisation during treatment with arimidex 1 and 10 mg, an arithmetic difference of 0.21% (95% confidence interval −4.99 to 4.57%) was found. However, if the analysis was repeated excluding the outlier patient (no. 9), *in vivo* aromatisation was suppressed by 96.0% during treatment with 1 mg and 98.6% during treatment with 10 mg, in which case the difference between the two situations became statistically significant ($P<0.01$). Calculating the ratio of the percentage aromatisation during treatment with arimidex 10 mg compared with 1 mg, this revealed a geometric mean value of 0.58 (95% confidence interval 0.29–1.15).

### *Plasma sex hormone levels*

Many patients achieved plasma levels of $E_2$ and $E_1$ during treatment that were below the sensitivity limits of the assay, in which case the value was given as the sensitivity limit (Table II and Figure 2).

Mean plasma level of $E_1$ was suppressed from 73.0 pmol $l^{-1}$ to 9.7 pmol $l^{-1}$ (mean suppression of 86.8%) and 9.8 pmol $l^{-1}$ (mean suppression of 86.5%) during treatment with anastrozole 1 mg and 10 mg respectively ($P<0.005$). Plasma levels of $E_2$ fell from a mean value of 17.7 pmol $l^{-1}$ before treatment to 2.8 pmol $l^{-1}$ and 2.9 pmol $l^{-1}$ (mean suppression of 84.0% and 83.5% respectively, $P<0.005$), and plasma levels of $E_1S$ decreased from a mean value of 387.2 pmol $l^{-1}$ to 25.3 pmol $l^{-1}$ and 16.9 pmol $l^{-1}$ (mean suppression of 93.5% and 95.7% respectively, $P<0.005$). No significant differences between plasma levels of any of the oestrogens during treatment with the two doses of anastrozole were seen.

Treatment with anastrozole had no significant influence on plasma levels of A (mean level of A before and during treatment with anastrozole 1 and 10 mg o.d. 4.1 nmol $l^{-1}$, 3.3 nmol $l^{-1}$ and 3.1 nmol $l^{-1}$ respectively).

**Table I** Effects of treatment with anastrozole 1 and 10 mg on peripheral aromatisation

| Patient | Pretreatment Arom.%[a] | Anastrozole (1 mg) Arom.%[a] | Anastrozole (1 mg) Supp.%[b] | Anastrozole (10 mg) Arom.%[a] | Anastrozole (10 mg) Supp.%[b] |
|---|---|---|---|---|---|
| 3 | 3.06 | 0.074 | 97.6 | 0.028 | 99.1 |
| 4 | 1.99 | 0.085 | 95.7 | 0.021 | 98.9 |
| 5 | 1.79 | 0.064 | 96.4 | 0.023 | 98.7 |
| 6 | 4.72 | 0.067 | 98.6 | 0.066 | 98.6 |
| 7 | 1.77 | 0.068 | 96.1 | 0.033 | 98.1 |
| 8 | 2.85 | 0.080 | 97.2 | 0.025 | 99.1 |
| 9 | 1.94 | 0.061 | 96.8 | 0.422 | 78.2 |
| 10 | 1.46 | 0.058 | 96.1 | 0.023 | 98.5 |
| 11 | 1.60 | 0.100 | 93.7 | 0.082 | 94.9 |
| 12 | 2.83 | 0.091 | 96.8 | 0.034 | 98.8 |
| Geom.[c] | 2.25 | 0.074 | 96.7 | 0.043 | 98.1 |
| 95% l.[d] | 1.73 | 0.065 | 95.6 | 0.022 | 96.1 |
| 95% u.[e] | 2.92 | 0.084 | 97.5 | 0.083 | 99.1 |

[a]Percentage of aromatisation. [b]Percentage suppression from baseline. [c]Geometrical mean value. [d]Lower limit of the 95% confidence interval of the mean. [e]Upper limit of the 95% confidence interval of the mean.

**Table II** Percentage suppression of plasma oestrogen levels during treatment with anastrozole 1 and 10 mg

| Drug dose | Oestrone 1 mg | Oestrone 10 mg | Oestradiol 1 mg | Oestradiol 10 mg | Oestrone sulphate 1 mg | Oestrone sulphate 10 mg |
|---|---|---|---|---|---|---|
| Patient | | | | | | |
| 2 | 93.1* | 88.3 | 86.6 | 79.3 | 95.6 | 97.1 |
| 3 | 77.1 | 82.2 | 62.6 | 65.3 | 58.4 | 61.8 |
| 4 | 77.7 | 82.5 | 79.4 | 76.1 | 93.0 | 82.8 |
| 5 | 92.8* | 92.8* | 93.5* | 93.5* | 96.4 | 99.5 |
| 6 | 91.7* | 91.7* | 85.0* | 85.0* | 92.4 | 96.3 |
| 7 | 89.6* | 89.6* | 83.1 | 85.2* | 94.8 | 97.4 |
| 8 | 83.8 | 82.0 | 89.5 | 93.4 | 91.0 | 96.7 |
| 9 | 76.9 | 80.2 | 74.1* | 74.1* | 92.9 | 97.2 |
| 10 | 75.7 | 79.4 | 79.0* | 78.0 | 96.0 | 97.4 |
| 11 | 83.6 | 75.7 | 71.8 | 58.8 | 89.6 | 90.9 |
| 12 | 93.2 | 93.4 | 93.5* | 93.5* | 98.1 | 94.9 |
| Geom.[a] | 86.8 | 86.5 | 84.0 | 83.5 | 93.5 | 95.7 |
| 95% l.[b] | 81.0 | 81.6 | 76.5 | 74.1 | 89.0 | 90.7 |
| 95% u.[c] | 90.7 | 90.1 | 89.1 | 89.5 | 96.1 | 98.0 |

*Values < sensitivity limit of the method. [a]Geometric mean value. [b]Lower limit of the 95% confidence interval of the mean. [c]Upper limit of the 95% confidence interval of the mean.

**Figure 1** Structure of anastrozole (Arimidex).

### *Plasma levels of anastrozole*

The mean plasma level of anastrozole was 37.7 ng $ml^{-1}$ (range 22.0–83.9 ng $ml^{-1}$) during treatment with a drug dose of 1 mg and 341.4 ng $ml^{-1}$ (range 160.0–644.0 ng $ml^{-1}$) during treatment with a dose of 10 mg daily.

### Discussion

Despite the fact that aminoglutethimide has been in clinical use for two decades and several other aromatase inhibitors for 5–10 years, many questions related to their biochemical action remain unaddressed. While the first study evaluating *in vivo* aromatase inhibition during treatment with aminoglutethimide reported the drug to inhibit the conversion of A to $E_1$ by about 98% (Santen *et al.*, 1978) and contemporary studies by us (MacNeill *et al.*, 1992; Jones *et al.*, 1992; Lønning *et al.*, 1991) and others (Reed *et al.*, 1990) have found aminoglutethimide, as well as novel aromatase inhibitors such as formestane and fadrozole, to inhibit *in vivo* aromatisation by about 90%, plasma oestrogens have been found to be sustained at 30–50% of their control levels

**Figure 2** Plasma levels of oestrone (**a**), oestradiol (**b**) and oestrone sulphate (**c**) in individual patients before treatment and following 4 weeks of treatment with anastrozole 1 mg and 10 mg. Dashed line gives the sensitivity limit of the assays.

in patients treated with these drugs (Santen *et al*., 1982*b*; Vermeulen *et al*., 1983; Dowsett *et al*., 1989,1990). Recent studies found the triazole drugs letrozole and vorozole to inhibit *in vivo* aromatisation by a mean of 98.5% and 93% respectively (Dowsett *et al*., 1995; Wall *et al*., 1993). While one group found letrozole to suppress plasma and urinary oestrogens by 90–95% (Masamura *et al*., 1994; Demers *et al*., 1993), others reported vorozole and letrozole to suppress plasma oestrogens by 55–90% (Iveson *et al*., 1993; Johnston *et al*., 1994), again revealing an internal inconsistency between the degree of aromatase inhibition and percentage of plasma oestrogen suppression. Such a difference could be due either to alternative (non-aromatase-dependent) oestrogen sources or lack of sensitivity of the radioimmunoassays used for plasma oestrogen measurement. Thus, there is a need to compare *in vivo* aromatase inhibition and plasma oestrogen suppression to develop the concept of aromatase inhibition in breast cancer treatment further.

This study was designed to determine *in vivo* aromatase inhibition and plasma oestrogen suppression during treatment with anastrozole, a novel aromatase inhibitor. Animal investigations (Plourde *et al*., 1995) suggest this drug to be a highly potent aromatase inhibitor, and preliminary studies in post-menopausal healthy women and breast cancer patients suggest the drug to be effective in suppressing plasma levels of $E_2$ (Plourde *et al*., 1994). To determine *in vivo* aromatisation, we used a sensitive and specific HPLC assay previously used by our group to evaluate different aromatase inhibitors (Jacobs *et al*., 1991). Plasma levels of $E_2$ and $E_1$ were measured with sensitive methods previously validated in our laboratories (Dowsett *et al*., 1987; Lønning *et al*., 1995). However, owing to low levels of these oestrogens (mean concentration of plasma $E_2$ and $E_1$ of about 20 and 75 pmol l$^{-1}$) in post-menopausal women (Lønning *et al*., 1995), it remains difficult to detect >90% suppression of these oestrogens from pretreatment levels. On the other hand, the oestrogen conjugate $E_1S$ is found in much higher concentrations than $E_2$ and $E_1$ in post-menopausal women. Plasma $E_2$, $E_1$ and $E_1S$ are at equilibrium (Lønning *et al*., 1990). Thus, as long as an aromatase inhibitor does not influence enzymes involved in the interconversion of these oestrogens (sulphatase or sulphotransferase) or interacts with oestrogen metabolism (Lønning and Kvinnsland, 1988), plasma $E_1S$ and the unconjugated oestrogens should be expected to be suppressed by the same percentage during treatment with aromatase inhibitors. To measure $E_1S$, we used a highly sensitive assay recently developed to determine plasma levels of this oestrogen in the very low range (Lønning and Ekse, 1995). Assuming a mean concentration of plasma $E_1S$ of about 400 pmol l$^{-1}$ in post-menopausal women, with a sensitivity of 2.7 pmol l$^{-1}$ this assay should be able to detect a 98–99% suppression of this plasma oestrogen conjugate.

This study reveals two important findings. First, it shows anastrozole, given as 1 mg or 10 mg o.d., to inhibit *in vivo* aromatisation by a mean value of 96.7% and 98.1%, respectively, and so to be one of the most potent aromatase inhibitors investigated so far. Secondly, treatment with anastrozole 1 and 10 mg o.d. suppressed plasma $E_1S$ by a mean value of 93.5% and 95.7% respectively. Therefore, our results revealed anastrozole at both doses administered to suppress plasma levels of $E_1S$ by a percentage close to the percentage aromatase inhibition. Of note, although without statistical significance, eight out of ten patients experienced a greater degree of $E_1S$ suppression when the higher dose of anastrozole was given. While we did not see a similar suppression of plasma $E_1$ and $E_2$, it is notable that many patients achieved plasma values of these oestrogens that were below the sensitivity limit of the methods. Thus, it is likely that we underestimated the percentage suppression of plasma $E_1$ and $E_2$. These data indicate that with the application of sufficiently sensitive assays, oestrogen suppression and aromatase inhibition are closely parallel in post-menopausal women.

While all patients experienced a suppression of *in vivo* aromatisation by >93.7% during treatment with anastrozole ⩾1 mg o.d., one patient experienced an inhibition of 78.2% only during treatment with 10 mg o.d. No definite explanation for this observation was found. It is noteworthy that this patient was the one experiencing the lowest plasma concentration of anastrozole (160 ng ml$^{-1}$) when treated with a dose of 10 mg daily, but this concentration was considerably higher than the highest plasma concentration observed among our patients when they were treated with a drug dose of 1 mg daily. All medication received in this trial was accounted for, and it is not likely that the patient may have failed to take her medication as prescribed.

The difference in aromatase inhibition between anastrozole given as 1 and 10 mg o.d. (arithmetic difference of 0.21%, difference of 1.4% between geometrical mean values) was not of statistical significance. However, eight out of ten patients achieved a better aromatase inhibition during treatment with the 10 mg dose compared with 1 mg. Excluding the one outlier patient (no. 9) from the analysis revealed a difference in aromatisation of statistical significance. Thus, there is evidence that most patients may achieve a somewhat better aromatase inhibition on 10 mg compared with 1 mg of anastrozole. However, the small magnitude of this difference and the fact that it was not accompanied by any significant difference in plasma oestrogen levels suggest that this difference may be of little clinical importance. Thus, our results and previous observations by others (Yates *et al.*, 1992), evaluating plasma $E_1$ and $E_2$ suppression with anastrozole single doses up to 60 mg, suggest that a dose escalation above 1 mg anastrozole once daily may not enhance plasma oestrogen suppression any further.

In conclusion, this study showed anastrozole given as 1 mg or 10 mg o.d. to be highly potent in inhibiting *in vivo* aromatisation and suppressing plasma oestrogens in post-menopausal breast cancer patients. Our results revealed anastrozole to suppress plasma levels of $E_1S$ by a percentage approaching the percentage aromatase inhibition, providing an internal consistency between alterations in the *in vivo* aromatisation and plasma oestrogen levels in these patients. The findings support the concept that effective aromatase inhibition is accompanied by a profound suppression of plasma oestrogens in post-menopausal breast cancer patients and refute a hypothesis of alternative sources of plasma oestrogens in such patients.

**Acknowledgements**

The authors are grateful to Dr N Ledonne and Dr A Selen (Zeneca Ltd.) for determining plasma anastrozole levels. This study was supported by grants from the Norwegian Cancer Society and the Cancer Research Campaign, UK. We are grateful to Mr D Ekse for his skilful technical assistance in determining plasma hormone levels.

### References

CASH R, BROUGH AJ, COHEN MNP AND SATOH PS. (1967). Aminoglutethimide (Elipten-CIBA) as an inhibitor of adrenal steroidogenesis. Mechanisms of action and therapeutic trial. *J. Clin. Endocrinol. Metab.*, **27**, 1239–1248.

COOMBES RC, GOSS P, DOWSETT M, GAZET JC AND BRODIE A. (1984). 4-hydroxyandrostenedione in treatment of postmenopausal patients with advanced breast cancer. *Lancet*, **2**, 1237–1239.

DEMERS LM, LIPTON A, HARVEY HA, KAMBIC KB, GROSSBERG H, BRADY C AND SANTEN RJ. (1993). The efficacy of CGS 20267 in suppressing estrogen biosynthesis in patients with advanced stage breast cancer. *J. Steroid Biochem. Mol. Biol.*, **44**, 687–691.

DOWSETT M, GOSS PE, POWLES TJ, HUTCHINSON G, BRODIE AMH, JEFFCOATE SL AND COOMBES RC. (1987). Use of the aromatase inhibitor 4-hydroxyandrostenedione in postmenopausal breast cancer: optimisation of dose and route. *Cancer Res.*, **47**, 1957–1961.

DOWSETT M, CUNNINGHAM DC, STEIN RC, EVANS S, DEHENNIN L, HEDLEY A AND COOMBES RC. (1989). Dose-related endocrine effects and pharmacokinetics of oral and intramuscular 4-hydroxyandrostenedione in postmenopausal breast cancer patients. *Cancer Res.*, **49**, 1306–1312.

DOWSETT M, STEIN RC, MEHTA A AND COOMBES RC. (1990). Potency and selectivity of the non-steroidal aromatase inhibitor CGS 16949A in postmenopausal breast cancer patients. *Clin. Endocrinol.*, **32**, 623–634.

DOWSETT M, JONES A, JOHNSTON SRD, JACOBS S, TRUNET P AND SMITH IE. (1995). *In vivo* measurement of aromatase inhibition be letrozole (CGS 20267) in post-menopausal patients with breast cancer. *Clin. Cancer Res.*, **1**, 1511–1515.

EVANS TRJ, SALLE ED, ORNATI G, LASSUS M, BENEDETTI MS, PIANEZZOLA E AND COOMBES RC. (1992). Phase I and endocrine study of exemestane (FCE 24304), a new aromatase inhibitor, in postmenopausal women. *Cancer Res.*, **52**, 5933–5939.

FOTSIS T AND ADLERCREUTZ H. (1987). The multicomponent analysis of oestrogens in urine by ion exchange chromatography and GC-MS-I. Quantitation of oestrogens after initial hydrolysis of conjugates. *J. Steroid Biochem. Mol. Biol.*, **28**, 203–213.

GRODIN JM, SIITERI PK AND MCDONALD PC. (1973). Source of estrogen production in postmenopausal women. *J. Clin. Endocrinol. Metab.*, **36**, 207–214.

IVESON TJ, SMITH IE, AHERN J, SMITHERS DA, TRUNET PF AND DOWSETT M. (1993). Phase I study of the oral nonsteroidal aromatase inhibitor CGS 20267 in healthy postmenopausal women. *J. Clin. Endocrinol. Metab.*, **77**, 324–331.

JACOBS S, LØNNING PE, HAYNES B, GRIGGS L AND DOWSETT M. (1991). Measurement of aromatisation by a urine technique suitable for the evaluation of aromatase inhibitors *in vivo*. *J. Enzyme Inhib.*, **4**, 315–325.

JOHNSTON SRD, SMITH IE, DOODY D, JACOBS S, ROBERTSHAW H AND DOWSETT M. (1994). Clinical and endocrine effects of the oral aromatase inhibitor vorozole in post-menopausal patients with advanced breast cancer. *Cancer Res.*, **54**, 5875–5881.

JONES AL, MACNEILL F, JACOBS S, LØNNING PE, DOWSETT M AND POWLES TJ. (1992). The influence of intramuscular 4-hydroxyandrostenedione on peripheral aromatisation in breast cancer patients. *Eur. J. Cancer*, **28A**, 1712–1716.

LIPTON A, DEMERS LM, HARVEY HA, KAMBIC KB, GROSSBERG H, BRADY C, ADLERCREUTZ H, TRUNET PF AND SANTEN RJ. (1995). Letrozole (CGS 20267). A phase I study of a new potent oral aromatase inhibitor of breast cancer. *Cancer*, **75**, 2132–2138.

LØNNING PE AND EKSE D. (1995). A sensitive assay for measurement of plasma oestrone sulphate in patients on treatment with aromatase inhibitors. *J. Steroid Biochem. Mol. Biol.*, **55**, 409–412.

LØNNING PE AND KVINNSLAND S. (1988). Mechanisms of action of aminoglutethimide as endocrine therapy of breast cancer. *Drugs*, **35**, 685–710.

LØNNING PE, DOWSETT M AND POWLES TJ. (1990). Postmenopausal oestrogen synthesis and metabolism: alterations caused by aromatase inhibitors used for the treatment of breast cancer. *J. Steroid Biochem. Mol. Biol.*, **35**, 355–366.

LØNNING PE, JACOBS S, JONES A, HAYNES B, POWLES T AND DOWSETT M. (1991). The influence of CGS 16949A on peripheral aromatisation in breast cancer patients. *Br. J. Cancer*, **63**, 789–793.

LØNNING PE, HELLE SI, JOHANNESSEN DC, ADLERCREUTZ H, LIEN EA, TALLY M, EKSE D, FOTSIS T, ANKER GB AND HALL K. (1995). Relations between sex hormones, sex hormone binding globulin, insulin-like growth factor-I and insulin-like growth factor binding protein-1 in post-menopausal breast cancer patients. *Clin. Endocrinol.*, **42**, 23–30.

MACNEILL FA, JONES AL, JACOBS S, LØNNING PE, POWLES TJ AND DOWSETT M. (1992). The influence of aminoglutethimide and its analogue rogletimide on peripheral aromatisation in breast cancer. *Br. J. Cancer*, **66**, 692–697.

MASAMURA S, ADLERCREUTZ H, HARVEY H, LIPTON A, DEMERS LM, SANTEN RJ AND SANTNER SJ. (1994). Aromatase inhibitor development for treatment of breast cancer. *Breast Cancer Res. Treat.*, **33**, 19–26.

PLOURDE PV, DYROFF M AND DUKES M. (1994). Arimidex: a potent and selective fourth-generation aromatase inhibitor. *Breast Cancer Res. Treat.*, **30**, 103–111.

PLOURDE PV, DYROFF M, DOWSETT M, DEMERS L, YATES R AND WEBSTER A. (1995). Arimidex: a new oral, once-a-day aromatase inhibitor. *J. Steroid Biochem. Mol. Biol.*, **53**, 175–179.

REED MJ, LAI LC, OWEN AM, SINGH A, COLDHAM NG, PUROHIT A, GHILCHIK MW, SHAIKH NA AND JAMES VHT. (1990). Effect of treatment with 4-hydroxyandrostenedione on the peripheral conversion of androstenedione to estrone and *in vitro* aromatase activity in postmenopausal women with breast cancer. *Cancer Res.*, **50**, 193–196.

SANTEN RJ, SANTNER S, DAVIS B, VELDHUIS J, SAMOJLIK E AND RUBY E. (1978). Aminoglutethimide inhibits extraglandular estrogen production in postmenopausal women with breast carcinoma. *J. Clin. Endocrinol. Metab.*, **47**, 1257–1265.

SANTEN RJ, SANTNER SJ, TILSEN-MALLETT N, ROSEN HR, SAMOJLIK E AND VELDHUIS JD. (1982*a*). *In vivo* and *in vitro* pharmacological studies of aminoglutethimide as an aromatase inhibitor. *Cancer Res.*, **42** (Suppl.), 3353s–3359s.

SANTEN RJ, WORGUL TJ, LIPTON A, HARVEY H AND BOUCHER A. (1982*b*). Aminoglutethimide as treatment of postmenopausal women with advanced breast carcinoma. *Ann. Intern. Med.*, **96**, 94–101.

SANTEN RJ, DEMERS LM, ADLERCREUTZ H, SANTNER S, SANDERS S AND LIPTON A. (1989). Inhibition of aromatase with CGS 16949A in postmenopausal women. *J. Clin. Endocrinol. Metab.*, **68**, 99–106.

VERMEULEN A, PARIDAENS R AND HEUSON JC. (1983). Effects of aminoglutethimide on adrenal steroid secretion. *Clin. Endocrinol.*, **19**, 673–682.

WALL EVD, DONKER TH, FRANKRIJKER ED, NORTIER HWR, THIJSSEN JHH AND BLANKENSTEIN MA. (1993). Inhibition of the *in vivo* conversion of androstenedione to estrone by the aromatase inhibitor vorozole in healthy postmenopausal women. *Cancer Res.*, **53**, 4563–4566.

YATES RA, DUKES M, DOWSETT M, DEBERARDINIS M, WILKINSON DM AND WILLIAMS AJ. (1992). Tolerability, pharmacokinetics and effect on serum oestradiol of ICI D 1033, a new aromatase inhibitor. *Clin. Pharm. and Ther.*, Abstract of the Fifth World Conference on Clinical Pharmacology and Therapeutics, Yokohama.